# Clinical feasibility of a quick response code-based digital self-reporting of medication adherence: results in patients on ticagrelor therapy from the APOLLO-QR observational study

**DOI:** 10.1093/ehjdh/ztaf056

**Published:** 2025-05-30

**Authors:** Bruno Francaviglia, Luca Lombardo, Bianca Pellizzeri, Federica Agnello, Rossella De Maria, Clelia Licata, Lorenzo Scalia, Florinda Bonanno, Mario Campisi, Antonio Greco, Piera Capranzano

**Affiliations:** Division of Cardiology, Policlinico Hospital, University of Catania, S. Sofia, Catania 95123, Italy; Division of Cardiology, Policlinico Hospital, University of Catania, S. Sofia, Catania 95123, Italy; Division of Cardiology, Policlinico Hospital, University of Catania, S. Sofia, Catania 95123, Italy; Department of Cardiology, Università degli Studi di Enna ‘Kore’, Enna, Italy; Division of Cardiology, Ospedale Umberto I, ASP 4 di Enna, Enna, Italy; Division of Cardiology, Policlinico Hospital, University of Catania, S. Sofia, Catania 95123, Italy; Division of Cardiology, Policlinico Hospital, University of Catania, S. Sofia, Catania 95123, Italy; Division of Cardiology, Ospedale Umberto I, ASP 4 di Enna, Enna, Italy; Division of Cardiology, Policlinico Hospital, University of Catania, S. Sofia, Catania 95123, Italy; Division of Cardiology, Policlinico Hospital, University of Catania, S. Sofia, Catania 95123, Italy; Division of Cardiology, Policlinico Hospital, University of Catania, S. Sofia, Catania 95123, Italy; Division of Cardiology, Policlinico Hospital, University of Catania, S. Sofia, Catania 95123, Italy

**Keywords:** Medication adherence, Telemonitoring, Ticagrelor therapy

## Abstract

**Aims:**

The APOLLO-QR (APPlying smartphOne for piLLs intake cOnfirmation by QR code reading) study assessed the congruence between a quick response (QR) code-based digital self-reporting and pill count in measuring medication adherence.

**Methods and results:**

The APOLLO-QR pilot, observational study prospectively included patients owning a smartphone accepting to undergo a home-telemonitoring of ticagrelor adherence by sending feedback of each pill intake through an email generated by framing a QR code placed on the medication packaging. Ticagrelor adherence was measured at 1 and 3 months by pill count allowing to calculate accuracy of the digital self-reporting in estimating drug adherence by assessing the correspondence between the number of received feedback emails and the number of pills taken from those prescribed. Among 109 patients, 30-day adherence to ticagrelor was 98.6 ± 2.6% as measured by pill count vs. 88.9 ± 10.4% as assessed by the number of feedback emails sent by the digital self-reporting, which provided an accuracy in estimating drug adherence of 90.1 ± 10.1%. Similar results were achieved at three months among the 95 patients (87.2%) continuing the study. Only nine patients (8.3%) missed sending four consecutive feedback emails of whom three (2.8%) had voluntarily discontinued ticagrelor within 1 month. A high patient satisfaction emerged from responses to a questionnaire showing that tested telemonitoring was consistently perceived as easy, convenient, and useful, although the need for more interactivity was suggested.

**Conclusion:**

The QR code-based self-reporting of pill intake showed a high accuracy in estimating medication adherence and yielded a good patient satisfaction, suggesting a potential for its clinical applicability.

## Graphical Abstract

**Graphical Abstract:**
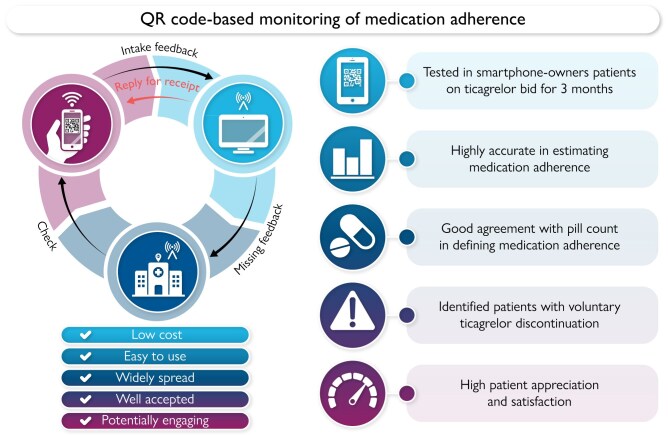
The Apollo-QR study included 115 patients on ticagrelor therapy to assess the clinical feasibility of a mobile health intervention to monitor home-therapy adherence consisting in sending feedback of medication intake by the smartphone reading of a quick response code label stuck on the pill box. This modality for digital self-reporting of pill intake was highly accurate and concordant in estimating medication adherence measured by the pill count, while being well accepted and appreciated by patients who were mostly satisfied by the monitoring strategy. Finally, the monitoring revealed useful for detecting ticagrelor disruption, with potential relevant clinical implications.

## Introduction

Novel pharmaceutical agents have markedly improved cardiovascular care. However, patient adherence to those therapies is often suboptimal, negatively impacting patient outcomes and increasing preventable healthcare costs.^1,2^ This unfavourable association has prompted the investigation of several methods to monitor adherence to prescribed home therapies including patient self-reporting of drug intake by several tools (i.e. diaries, interviews, questionnaires, and apps), electronic devices (i.e. pill bottles), artificial intelligence-based systems, pill count, and pharmacy claims.^3,4^ The electronic dispenser is considered the most reliable method that consists of a cap registering and wirelessly transmitting the exact time of patient interaction with medications by pill bottle opening, considered as a surrogate of drug intake.^5^ However, the clinical use of this electronic method is challenging due to its cost and demanding technical requirements. Conversely, patient self-reporting methods are easier and less expensive to be implemented, especially those based on mobile health (i.e. text messages and apps), which are currently recommended to improve medication adherence.^6,7^ However, the possibility for recall bias, due to the feedback collection deferred to the time of medication-taking, may affect their accuracy.^8^

In an attempt to overcome limitations of current methods of medication adherence monitoring we have proposed a simple, largely available and low-cost self-reporting method for real-time capturing of medication intake, consisting in transmitting digital feedback generated by the patient at every time the pill is taken by just reading with the smartphone an adhesive label with a quick response (QR) code placed on the medication packaging.^9^ Thus, the QR code reading, as the electronic cap opening, provides confirmation of the exact interaction of patient with medications with the potential advantages related to its technical simplicity and low cost.^10^ A preliminary study showed that the digital task of sending a QR code-based feedback was easily executed even by older patients who mostly were not using a smartphone.^9^ The APOLLO-QR (APPlying smartphOne for piLLs intake cOnfirmation by QR code reading) pilot study aimed to preliminarily investigate the clinical feasibility of the QR code-based real-time self-reporting of medication intake by assessing the accuracy of this low-cost telemonitoring in measuring medication adherence as compared with pill count and by collecting data on patients’ satisfaction.

## Methods

### Study design and population

The APOLLO-QR was a pilot, observational, non-interventional, prospective, single centre study, including patients undergoing percutaneous coronary intervention (PCI) in a tertiary centre, the Policlinico University Hospital, in Catania, Italy, from January 2023 to January 2024. The study population was screened among patients initiating therapy with ticagrelor 90 mg b.i.d. independently prescribed by the referral cardiologist discharging the patient. To avoid selection bias, all consecutive patients on ticagrelor discharged during working days in which study personnel was available for enrolment were screened. Only non-selected patients discharged when the research staff was unavailable were missed from screening. As the phase of ticagrelor initiation occurred before enrolment, the APOLLO-QR study explored the other two phases of medication adherence defined as implementation (i.e. number of doses taken out of those prescribed) and persistence (i.e. drug discontinuation) according to dedicated guidelines.^11^ Among medications prescribed at discharge, ticagrelor was selected to test the accuracy of the QR code-based digital self-reporting of medication adherence, as prescription and dispensation of this drug could be centrally managed in hospital, thus allowing for an accurate pill count.

Patients of age ≥ 18 years were enrolled in the study at the time of discharge if they owned a smartphone with an active internet plan, accepted to undergo ticagrelor adherence telemonitoring by reading a QR code placed on manufacturer’s packaging to generate a feedback email confirming each pill intake, and were able to execute this digital task as assessed by investigators by means of a test run. Patients were excluded if they did not own a smartphone or were not able to run the digital functions required for the study, or had inability to understand the study, or were not available for follow-up visits. The study was approved by the local Ethics Committee. All participants provided written informed consent.

### Study procedures

Initially, an email inbox (ApolloQR2021@gmail.com), accessible only to the study investigators, was set to collect patients’ digital feedback of pill intake. At the time of discharge, for enrolled patients, the following procedures were implemented: (i) to ensure privacy a unique encrypted email address (e.g. Patient 1 = 001apollo-QR@gmail.com) was created for each patient, from which feedback emails were sent; and (ii) an adhesive label with a QR code that encoded the ApolloQR2021@gmail.com inbox was attached to the manufacturer’s ticagrelor packaging supplied by hospital pharmacy. When the QR code was scanned using the patient’s smartphone camera, it generated an email that was sent to the aforementioned inbox, recording patient’s feedback at the time of each pill intake. The content of this email did not contain any personal identifiers or medical information; it simply codified as ‘pill taken’.

Study’s researchers daily monitored the study email inbox and manually logged, as a binary variable, the reception or the missing of each feedback in a dedicated spreadsheet designed to calculate for each patient the proportion of received intake feedback out of those expected based on doses prescribed. For ethical considerations, investigators contacted patients after four consecutive feedback entries were missing for detection of possible ticagrelor discontinuation. In case of verified voluntary discontinuation, it was recommended to consult the referral cardiologist. Besides these calls, no other types of interaction, such as daily reminders or alerts of medication intake, were used.

In response to each feedback email, an automatic delivery receipt, without mandatory reading, was sent to the encrypted patient inbox. Patients were instructed to refrain from sending delayed feedback from ticagrelor pill intake. Moreover, patients were advised not to use any other pill boxes for ticagrelor and to keep all blisters, including those that were empty or contained residual pills, in the provided manufacturer’s ticagrelor packaging, which was to be returned at on-site visits for pill count verification.

After enrolment, patients were monitored over three months. Follow-up visits were conducted at 24 ± 4 days and at three months. During these visits, patients underwent the clinical cardiological assessment scheduled as routine after PCI in our centre, and related to the study procedures, a ticagrelor pill count was performed by investigators to verify the congruence between received feedback emails and taken pills. Moreover, study investigators verbally questioned patients regarding their motivations for prematurely stopping the study or for missing feedback emails. Patients’ open-ended responses were subsequently clustered into reasons categories (reluctance, forgetfulness, technical or logistical issues). At 1-month visit, a patient satisfaction questionnaire was self-completed by patients before the clinical assessment was performed.

### Study endpoints and definitions

The Apollo-QR study assessed the implementation phase of ticagrelor adherence that was operatively measured by two ways: as the percentage of pills taken out of those prescribed (standard method), by pill count; and as the percentage of feedback of intake received out of those expected based on prescriptions (digital method), by telemonitoring. The primary endpoint of the study was the accuracy of the digital method in estimating ticagrelor adherence at 30 days compared with the pill count assumed as reference. The relative error in that estimation was calculated according to the following formula: ((number of pills taken from the provided packaging − number of feedback received)/number of pills taken from the provided box) × 100. Therefore, the accuracy percentage of tested monitoring method was calculated using the following formula: 100 − relative error.

Secondary study endpoints related to the implementation phase of ticagrelor adherence included: the concordance between pill count and telemonitoring in defining a patient as adherent to ticagrelor by using two adherence cut points, equal or higher than the minimum value of adherence measured by pill count and ≥80%, a cut-point commonly used in literature^3^; the accuracy of the telemonitoring in estimating ticagrelor adherence at 90 days.

Moreover, a secondary study objective related to the persistence phase of medication adherence aimed to detect ticagrelor discontinuation defined as four consecutive doses not taken.

Another study objective was to assess patient satisfaction measured through a researcher developed questionnaire in which responses were rated on a five-point Likert scale to collect patient opinion on telemonitoring about perceived health usefulness, usability, and privacy respect.

Finally, a subgroup analysis stratified by optimal or suboptimal responsiveness to the QR code-based self-reporting at one month was performed. Responsiveness to telemonitoring was defined as optimal or suboptimal when the accuracy percentage was above or below the first quartile, respectively. This analysis aimed to assess differences between groups in clinical characteristics and reasons for missing feedback emails to understand further telemonitoring system improvements. Moreover, potential variables independently associated with patients’ response to telemonitoring were identified.

### Statistical analysis

As the APOLLO-QR qualified as a pilot study, a precision-based sample size was calculated. A sample of 91 patients was estimated by assuming a margin of error of 10% in accuracy and specifying a 95% confidence interval (CI). A margin of uncertainty of plus or minus 10% was considered reasonably tolerable and not impacting conclusions of this exploratory analysis.

Continuous variables are presented as means ± standard deviations and median (interquartile range) and were compared using the Student’s *t*-test. Categorical variables are presented by absolute counts and percentages and were compared using the χ^2^ or Fisher’s exact tests, as appropriate.

The concordance between pill count and telemonitoring in defining a patient as adherent to therapy by using different medication adherence cut points was assessed by the Kappa index. This latter was calculated by ratio between the number of patients defined adherent with both methods and the total number of patients. The strength of the agreement was defined as very good, good, moderate, and low for Kappa values of 0.81–1.00, 0.61–0.80, 0.41–60, and <0.41, respectively.^12^

To evaluate reliability, the internal consistency of responses to questionnaire was assessed by computing a Cronbach’s α coefficient. Values above 0.80 indicate good internal consistency.^13^

The multivariate binary logistic regression analysis was used to evaluate the association of potential explanatory variables with suboptimal responsiveness to telemonitoring. Results are reported as odds ratio (OR) and the corresponding 95% CI. All *P*-values of <0.05 were considered as statistically significant. Statistical analyses were performed using the Statistical Package for Social Sciences, version 22 (SPSS Inc., Chicago, IL, USA).

## Results

### Study population

**Figure 1:**
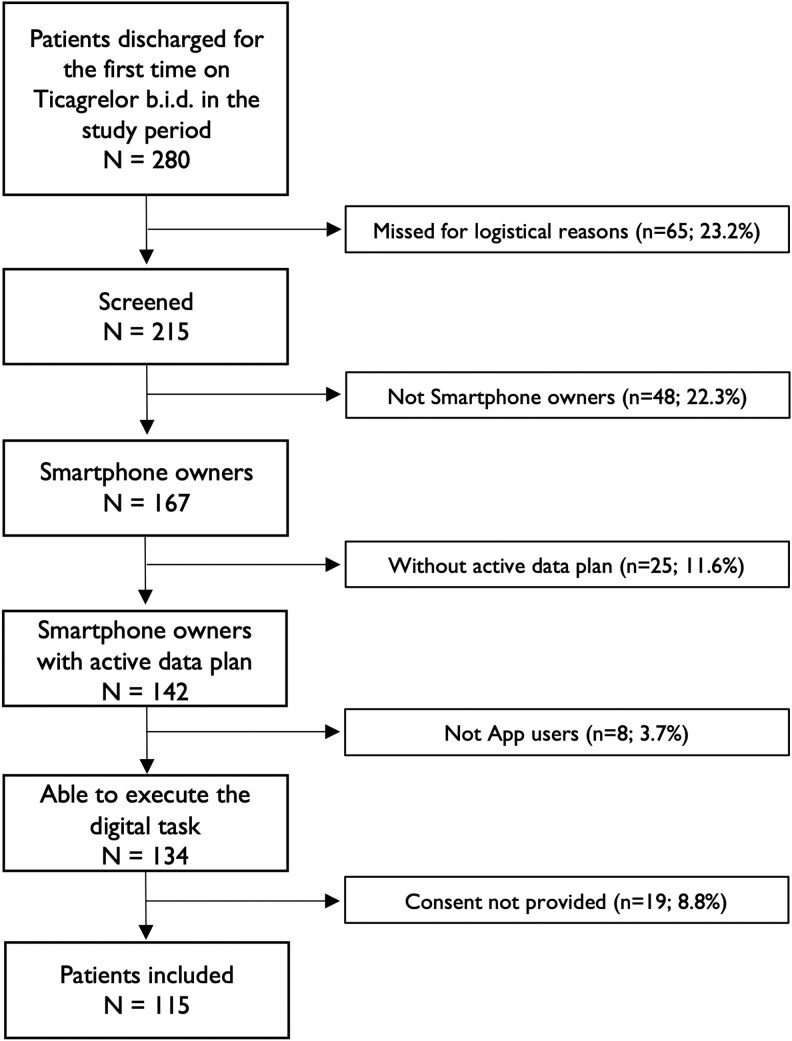
Study population flow-chart.

The study population flow-chart is illustrated in *Figure 1*. Among a total of 215 screened patients on ticagrelor at discharge, 81 (37.7%) had exclusion criteria: 48 (22.3%) were not smartphone owners, 25 (11.6%) had a smartphone without internet plan, and 8 (3.7%) were not apps users. Among patients with inclusion and without exclusion criteria (*n* = 134), 19 (14.2%) were not willing to participate in the study, and thus a final cohort of 115 patients (85.8%) signed the informed consent and was included in the study. Demographic and clinical characteristics of those included patients are shown in *Table 1*, with no participants having missing data for each variable. Among study population, the mean age was 59.0 ± 10.6 years, and the proportion of patients aged ≥65 was 29.6%. Only 27.0% were in retirement status, which could be a potential confounder in the association of different measured variables with response to telemonitoring.

**Table 1:** Study population characteristics

	Overall population
	*n* = 115
Age (years)	59.0 ± 10.6
	61 (50–66)
Female, *n* (%)	13 (11.3)
Body mass index	28.0 ± 6.0
	27.2 (24.6–29.7)
School education, *n* (%)	
Middle	42 (36.5)
High	41 (35.7)
Graduation	22 (19.1)
Low	10 (8.7)
Previous or current job, *n* (%)	
Workman	27 (23.5)
Professional/manager	25 (21.7)
Employee/teacher	23 (20.0)
Artisan	12 (10.4)
Merchant	11 (9.6)
Service industry worker	8 (7.0)
Household	6 (5.2)
Farmer	3 (2.6)
Practicing hobbies, *n* (%)	51 (44.3)
Retired	31 (27.0)
Income, *n* (%)	
Middle	58 (50.4)
Low	42 (36.5)
High	15 (13.0)
No apps users, *n* (%)	24 (20.9)
Apps used, *n* (%)	
Social	90 (78.3)
News	73 (63.5)
Gaming	46 (40.0)
Home-banking	42 (36.5)
Movie/sport/TV	20 (17.4)
Health, fitness, travel, others	15 (13.0)
Risk factors, *n* (%)	
Current or prior smoker	84 (73.0)
Hypertension	64 (55.7)
Hyperlipaemia	59 (51.3)
Diabetes	21 (18.3)
Clinical presentation, *n* (%)	
ST-elevation myocardial infarction	68 (59.1)
Non-ST-elevation myocardial infarction	35 (30.4)
Unstable angina	12 (10.4)
Comorbidities	44 (38.3)
Left ventricular ejection fraction %	47.9 ± 8.1
	50 (42–55)
No. of drugs at discharge	
Once a day	5.22 ± 1.5
	5 (4–6)
Twice a day	1.71 ± 0.98
	1 (1–2)

Continuous variables are presented as means ± standard deviations and medians (interquartile range).

### Study outcomes on implementation phase of medication adherence

**Figure 2:**
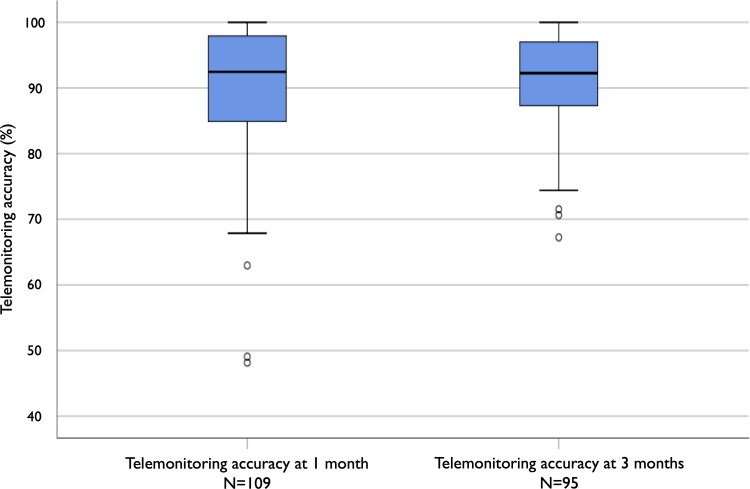
Accuracy of telemonitoring in assessing medication adherence at 1 and 3 months. The central box represents the values from the lower to upper quartile (25th–75th percentile). The middle horizontal line represents the median (50th percentile). The vertical line extends from the minimum to the maximum value within 1.5 box heights from the top or bottom of the box. Outliers are displayed as separate points.

Endpoint results at one month were obtained from a total of 109 patients as six (5.2%) abandoned the study within one week after inclusion, of whom three patients due to technical issues with their old and entry-level smartphone, while the other three were just reluctant to send feedback emails without providing specific reasons. The mean value of adherence to ticagrelor therapy at one month was 98.6 ± 2.6% as measured by pill count compared with 88.9 ± 10.4% as assessed by the number of QR code-based feedback emails sent by patients. Therefore, the study telemonitoring estimated drug adherence at one month with mean and median accuracy values of 90.1 ± 10.1% and 92.5% (84.3–97.9%), respectively (*Figure 2*). The 1-month accuracy value of the QR code-based self-reporting was <50% (minimum value 48.2%) only in two patients (1.8%), while in all the other patients (*n* = 107) was >60%: between 61% and 80% in 11 (10.1%), 81% and 90% in 30 (27.5%), and 91% and 100% in 66 (60.5%) subjects.

The strength of the agreement between pill count and digital self-reporting in defining a patient as adherent by using cut points of medication adherence ≥ 87% (the minimum value of ticagrelor adherence measured by pill count) and ≥80% was good (kappa 0.67) and very good (kappa 0.86), respectively.

**Figure 3:**
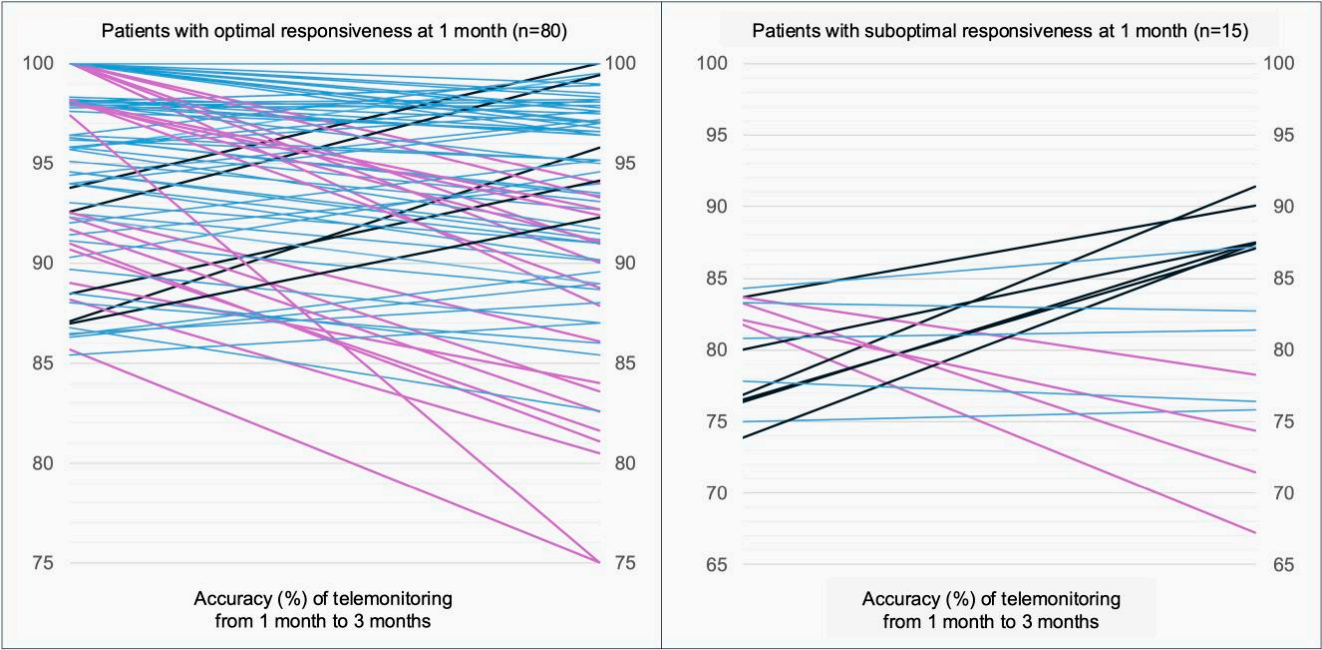
Change in telemonitoring accuracy in assessing medication adherence from 1 to 3 months in each patient with and without optimal responsiveness. Patients maintaining a stable accuracy with absolute change within 5% are indicated with blue lines; patients with a >5% absolute reduction or increase in accuracy are indicated in pink or black lines, respectively.

Among 109 patients reaching the 1-month follow-up, 95 (87.2%) of them decided to continue the study and all of them persisted up three months. Of remaining 14 patients, four withdrew the study because they found the system annoying or not useful, while the other 10 subjects were just reluctant to continue even though they favourably considered the QR code-based self-reporting. At three months, the mean value of adherence to ticagrelor measured by pill count was 98.6 ± 1.9% compared with 89.6 ± 8.0% as based on numbers of feedback emails received. Therefore, at three months, the tested telemonitoring modality estimated ticagrelor adherence with mean and median accuracy values of 90.8 ± 7.4% and 92.3% (87.2–97.0%), respectively (*Figure 2*). From one to three months, the telemonitoring accuracy values maintained substantially stable in most (65.3%) patients continuing over the entire follow-up period, significantly decreased in 22 (23.2%) patients, while markedly improved in 11 (11.6%) subjects (*Figure 3*).

### Study outcomes on persistence phase of medication adherence

Over one month nine patients (8.3%) were contacted by the investigators due to four consecutive missed feedback emails for at least one time, leading to 10 calls to verify drug intake. Ticagrelor discontinuation was found only in three patients (2.8%) who had voluntarily interrupted the drug because of mild bleeding, while remaining six patients (5.5%) were regularly taking ticagrelor, but they missed sending feedback emails. All nine patients continued the study after the call, but the six patients missing the four consecutive feedback emails despite taking medications stopped the study at 1 month. The three patients who had discontinued therapy restarted sending feedback emails when ticagrelor was resumed as indicated by referral cardiologists. At 1-month visit, no patients were found to have discontinued the drug besides those three identified by missed feedback emails before the visit in person occurred.

From one to three months, no patients had missed at least four consecutive feedback emails, and none had discontinued the drug.

### Study outcomes on patient satisfaction

Responses to individual questionnaire items showed high mean scores (ranging from 4.53 to 4.91 out of 5), indicating a good satisfaction degree (*Table 2*). The coefficient of internal consistency of responses was 0.89 showing good reliability.

**Table 2:** Patient satisfaction questionnaire

Questions	Score (out of 5) Mean ± SD	Var S
Q1—I believe that this therapy monitoring is beneficial for my health	4.75 ± 0.83	0.688
Q2—I felt reassured to be monitored in my therapy	4.76 ± 0.72	0.517
Q3—I found this therapy monitoring to be appropriate for my clinical condition	4.85 ± 0.65	0.423
Q4—I found it easy to execute the required digital task	4.91 ± 0.50	0.251
Q5—I found this therapy monitoring convenient	4.82 ± 0.71	0.503
Q6—I believe that this therapy monitoring could be improved by adding daily reminder and alert of pill intake, and other health notifications	4.85 ± 0.57	0.330
Q7—I did not find this therapy monitoring intrusive to my privacy	4.73 ± 0.65	0.419
Q8—I did not find annoying to perform the required digital task	4.79 ± 0.73	0.538
Q9—I would recommend this this therapy monitoring to other patients	4.72 ± 0.65	0.424
Q10—Overall, I am satisfied with this therapy monitoring	4.53 ± 0.76	0.585

SD, standard deviation; Var S, sample variance.

Question 4 on system usability had the highest mean score (4.91) and the lowest variance (0.251), indicating a consistent perception of ease of use among participants. Items evaluating telemonitoring perceived health usefulness (Q1 and Q2), its appropriateness for the clinical condition (Q3), convenience (Q5), privacy respect (Q7), and the willingness to recommend the system (Q9) all had mean scores ≥ 4.7 and relatively low variances, suggesting that patients were overall positive about both system functionalities and privacy preservation. Regarding Q6, which asked patients’ opinion about the potential utility of system enhancements with daily reminders and alerts of pill intake, as well as with additional health notifications, showed a mean of 4.85 with a variance of 0.3, suggesting patients’ appreciation for a more comprehensive and interactive monitoring of medication adherence.

The slightly lower score observed for Q10 on general patient satisfaction compared with that of single items may reflect the fact that when judging the overall perception patients may have weighed also some perplexities that were not captured in the other items or suggestions for improvements.

### Subgroup analysis

Demographic and clinical characteristics of patients reaching the 1-month endpoint and subgroups stratified according to optimal or suboptimal responsiveness to the drug adherence monitoring are illustrated in *Table 3*. No significant differences between groups were observed. In the multivariable model including variables shown in *Table 3*, only the low level of school education emerged as independent predictor of suboptimal responsiveness (OR 4.18; 95% CI 1.04 −16.9; *P* = 0.04).

**Table 3:** Characteristics of overall population reaching 1-month endpoint and in subgroups stratified across responsiveness

	Overall population	Optimal responsiveness	Suboptimal responsiveness	*P*-value
	*n* = 109	*n* = 81	*n* = 28	
Age (years)	59.0 ± 10.4	59.4 ± 9.5	57.9 ± 12.8	0.52
	61 (50.5–65.5)	61 (51–65)	59 (46.5–66.7)	
Female, *n* (%)	12 (11.0)	9 (11.1)	3 (10.7)	1.00
Body mass index, kg/m^2^	27.6 ± 4.6	28.0 ± 4.9	26.6 ± 3.2	0.16
	27.2 (24.9–29.6)	27.3 (24.0–30.0)	26.6 (24.2–28.8)	
School education, *n* (%)				
Middle	40 (36.7)	32 (39.5)	8 (28.6)	0.30
High	39 (35.8)	28 (34.6)	11 (39.3)	0.65
Graduation	21 (19.3)	17 (21.0)	4 (14.3)	0.62
Low	9 (8.3)	4 (4.9)	5 (17.9)	0.05
Previous or current job, *n* (%)				
Skilled workers	67 (61.5)	50 (61.7)	17 (60.7)	1.00
Unskilled workers	42 (38.5)	31 (38.3)	11 (39.3)	
Practicing hobbies (%)	48 (44.0)	37 (45.7)	11 (39.3)	0.71
Retired (%)	31 (28.4)	22 (27.2)	9 (32.1)	0.79
No app users, *n* (%)	21 (19.3)	17 (21.0)	4 (14.3)	0.62
Income, *n* (%)				
Middle/high	69 (63.3)	50 (61.7)	19 (67.9)	0.72
Low	40 (36.7)	31 (38.3)	9 (32.1)	

Continuous variables are presented as means ± standard deviations and medians (interquartile range).

Among the subgroup (*n* = 28) with suboptimal responsiveness at 1 month, main reasons for poor participation included reluctance in 12 patients (42.9%), technical or logistical issues in nine (32.1%), and forgetfulness in seven (25%) subjects. The missing feedback by patients showing an optimal responsiveness but not reaching a telemonitoring accuracy of 100% (*n* = 64) were mostly attributed to technical or logistical issues, while sporadic forgetfulness was reported by five patients (7.8%).

Only 21% of patients (*n* = 6) with 1-month suboptimal responsiveness missed to send four consecutive feedback emails for at least one time requiring seven calls, and all of them were found to assume ticagrelor regularly. Conversely, only 3.7% (3 out of 81) of patients have missed four consecutive feedback within the optimal responsiveness subgroup, and all of them had voluntarily discontinued ticagrelor.

After one month, 80 (98.8%) and 15 patients (53.6%) with optimal and suboptimal responsiveness, respectively, continued the study over three months. At three months, among patients showing a 1-month suboptimal response, a total of six (40%) significantly improved, four (26.7%) patients yielded a worse accuracy and the remaining maintained stable. Among those with optimal responsiveness at one month, accuracy further improved in five (6.3%) patients, while significantly decreased in 18 (22.5%) patients (*Figure 3*).

## Discussion

The Apollo-QR study firstly showed that the QR code-based digital self-reporting of pill intake provided a high accuracy in estimating home-therapy adherence compared to pill count and was well accepted by patients (*Graphical Abstract*).

The favourable results shown in this study are even more promising if considering that tested digital self-reporting of medication intake, based on sending email by QR code reading, had very basic functionalities, which often, in less advanced smartphones, required several digital steps to send the feedback. Indeed, the email was used as a free-access digital platform to receive the QR code-generated records as funding for engineering specific software was not available. Moreover, the good performance of tested telemonitoring was achieved without daily automatic reminders and alerts of pill intake, or other types of health notifications to enhance patient engagement. It is reasonable to expect better results by implementing the smartphone QR code reading in a digital platform using advanced encrypted and low-latency data transmission protocols to manage the interaction with patients, caregivers, and physicians by automatically reminding pill intake, updating the pill count after each feedback, alerting of either missing or discrepant feedback, and sending medical notifications.

A digital monitoring system could include several modalities for the patient to report feedback of pill intake. Indeed, records of medication consumption may be generated automatically by just opening electronic medications bottles or may be self-reported by patients through several digital methods, including annotations in apps or text messages.^3,4^ Compared to electronic devices in which it is assumed that every bottle opening corresponds with medication intake, in our tested modality, the QR code reading would replace the electronic cap opening while being costless, technically simpler and widely spread. However, compared to electronic devices, which passively register data on medication use, the QR code-based transmission of pill intake requires the active code reading by patients. This aspect may potentially reduce the correlation between the QR code-based feedback and medication adherence when a patient assumes the pill but forgets to scan the QR code. However, the QR code-based monitoring showed a high accuracy in estimating medication adherence. Moreover, the contribution of forgetfulness of reading the QR code on responsiveness to monitoring was minimal. This observation was probably due to both, the evocative image well visible on the pill box and the time concomitance between the pill intake and the action of framing the QR code. The latter feature differentiates the QR code-based feedback from other digital self-reporting strategies (i.e. apps) that do not act as packaging reminders. Mobile health apps have shown promising effects on medication adherence, including in patients with cardiovascular disease.^14,15^ However, this tool may be less suitable for patients with limited technological literacy. Additionally, because data entry in apps can be deferred from pill intake, there is an inherent risk of memory biases and transcription errors, which may reduce the accuracy of adherence reporting.^8,16^ In this regard, the concordance in assessing medication adherence between self-reporting methods and other monitoring modalities (i.e. electronic devices or pill count) largely varies across studies, which rarely reported good concordance levels such as those observed between the QR code-based digital self-reporting and pill count.^17^ Indeed, it might be hypothesized that the real-time feedback transmission by the QR code framing at the time the patient interacts with the pill box may potentially offer the opportunity for a more accurate and precise correlation with drug use than that provided by other digital self-reporting methods.

Finally, an intriguing finding observed in the Apollo-QR study is that only the low level of school education was found to be independently associated with suboptimal responsiveness. It is likely that unmeasured psychological and sociological factors may have an impact on active participation to healthcare initiative, potentially underlying the reluctance in accomplishing the monitoring digital task.^18^ It remains unknown whether possible reasons of reluctance may have been overwhelmed by a more interactive modality. Indeed, patients consistently believed that a medication adherence monitoring would improve with the use of several interaction tools such as daily automatic reminders and alerts of pill intake, motivational messages, educational information and medical notifications. Most of these latter have been shown to positively impact on medication adherence leading recent European guidelines to strongly support the use of mobile health strategies to improve adherence to medical therapy and healthy lifestyles.^7,19–21^ However, no specific tools were recommended, and the implementation of different mobile health approaches in a single patient-centred full-digital system with an interactive interface providing also a real-time feedback of drug intake might represent an optimal option to monitor and aid home-therapy adherence.^22^ The QR code integrated into the manufacturer’s medication packaging would not only be a visual reminder to send the feedback of drug intake but could leverage the use of that packaging, rather than electronic devices, as a nudge for medication adherence and an innovative tool for patient engagement in the healthcare process. Indeed, it has been shown that drug reminder packaging increases the percentage of drug intake.^23^ Nevertheless, possible options to generate the feedback of therapy intake should be individualized according to patients’ clinical characteristics, needs, habits, and attitudes. In this regard, the present study showed that most patients are well-disposed towards self-reporting modalities requiring their active participation and commitment and, also, they appreciate and feel reassured in being observed and followed. However, study findings also suggest the need for more attractive monitoring systems that could assist the overall therapeutic management. The role for non-financial incentives and rewards on prompting adherence to therapies, healthy lifestyle behaviours, and healthcare initiatives is also worthy to be assessed in future studies.

### Study limitations

Study results should be interpreted considering several limitations. First, a relatively small sample from a single centre was included. However, the study population is representative of that treated in other tertiary centres across countries, as shown in real-world registries, despite the slightly younger mean age of patients enrolled in the Apollo-QR study.^24–26^ This latter difference may be due to the relatively lower use of smartphone among elderly.^9^ However, a preliminary study showed the suitability of the digital task consisting in sending a QR code-based feedback among an elderly population.^9^ Moreover, the smartphone use is progressively increasing among elderly worldwide. Second, a 10% margin of error was assumed for sample size calculation. However, considering the achieved 90% mean accuracy, a value within the range of plus or minus 10% may be still considered reasonably high. Third, it should be considered that an appreciable percentage of patients were excluded from the study due to lack of technology, while small proportions were unwilling to be enrolled, or abandoned the study within one week, thus restricting applicability of tested telemonitoring. This latter limitation may be attenuated by the wider spread of technologies along with the implementation of more advanced and attractive features in telemonitoring systems. Fourth, the feasibility assessment was performed only with ticagrelor, which was selected as a medication to test the proposed home-therapy telemonitoring as its prescription and dispensation in hospital allowed for a simpler and more accurate pill count. However, it should be recognized that pill count has some drawbacks as it is susceptible to patient manipulation and thus further validation studies might compare the QR code-based self-reporting with more objective measures of medication adherence. Finally, the impact on medication adherence of the digital self-reporting remains undetermined as it was beyond the scope of the present investigation. However, the medication adherence monitoring allowed for detection of not clinically indicated ticagrelor discontinuation. Also, it is unknown if telemonitoring somehow impacted on the observed high ticagrelor adherence.

## Conclusions

The Apollo-QR pilot study provided preliminary promising data on the feasibility in clinical practice of the QR code-based digital self-reporting of medication adherence. Indeed, the high level of accuracy in estimating medication adherence, the good patient satisfaction, along with favourable features of the QR code (i.e. low cost and widespread availability), would suggest a potential for clinical applicability of the assessed mobile health strategy. Therefore, study findings pave the way for future technology development and larger studies with longer follow-up assessing clinical effectiveness of the QR code-based feedback implementation in full-digital systems for monitoring and support medication adherence.

## Data Availability

The data underlying this article will be shared on reasonable request to the corresponding author.
